# Physical Activity Trends Among Adults in a National Mobile Health Program: A Population-Based Cohort Study of 411,528 Adults

**DOI:** 10.1093/aje/kwac193

**Published:** 2022-11-07

**Authors:** Gregory Ang, Sarah Martine Edney, Chuen Seng Tan, Nicole Lim, Jeremy Tan, Falk Müller-Riemenschneider, Cynthia Chen

**Affiliations:** Department of Statistics and Data Science, National University of Singapore, Singapore; Saw Swee Hock School of Public Health, National University of Singapore and National University Health System, Singapore; Saw Swee Hock School of Public Health, National University of Singapore and National University Health System, Singapore; Yong Loo Lin School of Medicine, National University of Singapore, Singapore; Health Promotion Board, Ministry of Health, Singapore; Health Promotion Board, Ministry of Health, Singapore; Saw Swee Hock School of Public Health, National University of Singapore and National University Health System, Singapore; Yong Loo Lin School of Medicine, National University of Singapore, Singapore; Berlin Institute of Health, Charité University Medical Centre, Berlin, Germany; Saw Swee Hock School of Public Health, National University of Singapore and National University Health System, Singapore; Schaeffer Center for Health Policy and Economics, University of Southern California, USA; Department of Non-Communicable Disease Epidemiology, The London School of Hygiene & Tropical Medicine, UK

**Keywords:** physical activity, mHealth, mobile health, nationwide program, regression discontinuity design, difference-in-difference

## Abstract

Physical inactivity is a global public health challenge, and effective, large-scale interventions are needed. We examined the effectiveness of a population-wide mobile health (mHealth) intervention in Singapore, National Steps Challenge Season 3 (NSC3) and 2 booster challenges (Personal Pledge and Corporate Challenge). The study includes 411,528 participants. We used regression discontinuity design and difference-in-difference with fixed-effects regression to examine the association of NSC3 and the additional booster challenges on daily step counts. Participants tended to be female (58.5%), with an average age of 41.5 years (standard deviation, 13.9) and body mass index (weight (kg)/height (m)^2^) of 23.8 (standard deviation, 4.5). We observed that NSC3 was associated with a mean increase of 1,437 steps (95% confidence interval (CI): 1,408, 1,467) per day. Enrollments in Personal Pledge and Corporate Challenge were associated with additional mean increases of 1,172 (95% CI: 1,123, 1,222) and 896 (95% CI: 862, 930) steps per day, respectively. For NSC3, the associated mean increase in the step counts across different sex and age groups varied, with greater increases for female participants and those in the oldest age group. We provide real-world evidence suggesting that NSC3 was associated with improvements in participants’ step counts. Results suggest NSC3 is an effective and appealing population-wide mHealth physical activity intervention.

## Abbreviations

BMI: body mass index
CI: confidence interval
DID: difference-in-difference
IQR: interquartile range
mHealth: mobile health technologies
NSC: National Steps Challenge
NSC3: National Steps Challenge Season 3
RDD: regression discontinuity design

The health benefits of physical activity are well-established (1, 2). World Health Organization (WHO) guidelines recommend that adults engage in 150–300 minutes of moderate-intensity physical activity, or 75–150 minutes of vigorous-intensity physical activity, per week (3). Yet currently, over a quarter (27.5%) of the global adult population does not meet these guidelines (4). In response, the WHO has released a Global Action Plan on Physical Activity (GAPPA), which sets a target of reducing global levels of physical inactivity by 15% by 2030 (5). Achieving this target will require effective physical activity interventions that can be implemented on population-wide scales.

Mobile health technologies (“mHealth”) offer a potential platform to deliver population-wide interventions and have demonstrated efficacy within randomized controlled trials (RCTs). However, to translate these improvements to population health, effective interventions need to be scaled up and delivered to large numbers of people within real-world contexts (6).

Examples of interventions implemented on population-wide scales in real-world contexts are scarce (7), and challenges in rigorously evaluating them mean that evidence of their effectiveness is limited (8). Although RCTs are considered the gold standard for studying causal relationships (9, 10), they are not always possible or practical to implement (11). In their conventional form, RCTs randomize participants into 2 groups, one that receives the intervention and one that does not. Policy makers and public health practitioners may hence consider it unethical to deny a potentially beneficial intervention to half the population (12).

Quasi-experimental study designs that use observational data to estimate causal effects are considered the “next-best” approach for rigorous evaluation (13). However, few examples of such approaches are applied to physical activity programs (14, 15) or mHealth (16). As such, evidence of the impact of large-scale mHealth interventions implemented in real-world contexts remains largely unknown. This is a significant gap given the potential health benefits of population-wide increases in physical activity.

The National Steps Challenge (NSC) is a population-wide mHealth physical activity intervention conducted in Singapore yearly since 2015. Previous evaluation of NSC has shown strong uptake of and engagement with the intervention, and some preliminary evidence of effectiveness (17). However, a formal rigorous evaluation of the effectiveness of NSC has not yet been conducted. This study evaluates the effectiveness of the NSC Season 3 (NSC3) on physical activity. The study also investigates whether this differed across sex and age groups and assesses the additional impact of 2 booster challenges embedded within.

## METHODS

### Study design

Quasi-experimental study design using regression discontinuity and difference-in-difference with fixed-effects regression was used to examine the association of NSC3, and the additional booster challenges (Personal Pledge and Corporate Challenge), with daily step counts.

### Setting and participants

Singapore residents aged 17 years or older were eligible and were recruited via print posters, social media, and public roadshows (18). The present study includes participants who signed up between September 28, 2017, and March 31, 2018, and provided at least 1 day of valid step counts (defined as a day with any steps recorded).

Participants were excluded if their demographic characteristics (sex, age) or height and weight were missing or implausible (i.e., weight of >300 kg, height of >220 cm).

#### Intervention.

Delivered via an activity tracker and app, NSC3 consists of a 5-month intervention period with optional, embedded booster challenges. The design of NSC3 was guided by behavioral science, including gain-framed financial incentives for reaching predefined daily step targets, nudging via reminders and real-time feedback on physical activity levels, and prior commitments to daily physical activity step targets (19). The intervention also targeted social norms (20) toward physical activity via a nationwide marketing campaign and within the team-based Corporate Challenge. The tiered system of daily step targets was intended to provide participants with an incentive to make even small changes to their daily physical activity (Web Table 1, available at https://doi.org/10.1093/aje/kwac193). Gain-framing was used (i.e., sure-win vouchers) to ensure that there would be few barriers to joining the intervention (Web Table 2). This was considered particularly important as the NSC is delivered by the government of Singapore’s health-promoting agency (Health Promotion Board) and is intended to reach as many residents of Singapore as possible. The monetary rewards were intended to be appealing without being overly coercive—and Health Points (that can be exchanged for vouchers; Web Table 2) were considered most appropriate, as residents can earn these points for a range of different health-promoting activities (e.g., purchasing healthy foods). Full details of the main NSC3 intervention and the 2 nested booster challenges are included below and in Web Appendix 1.

#### NSC3 main intervention.

The NSC3 main intervention ran from October 28, 2017, to March 31, 2018 (days 0–154). During these 155 days, participants used their own tracker (i.e., wearable, smartphone) or a free NSC3 step tracker to monitor their daily step counts.

#### Personal Pledge.

Once participants achieved all the rewards in the main intervention, they could enroll in the optional Personal Pledge, where they pledged to achieve a daily steps target for a stipulated number of days, ranging from 15 to 120 days, to win special prizes worth up to $70 (Web Table 3). The more days participants pledged and achieved their pledge, the greater the value of the prizes. Personal Pledge ran for the entirety of the main intervention period (i.e., October 28, 2017, to March 31, 2018, days 0–154). Pledge completers were defined as NSC3 participants who completed their pledge, while Pledge noncompleters were defined as those who enrolled but did not complete their pledge. Non–Pledge participants were defined as those who did not enroll in Personal Pledge, either because they were not eligible or because they chose not to enroll.

#### Corporate Challenge.

Participants could enroll in the Corporate Challenge if their company registered in this team-based step challenge. The Corporate Challenge ran from January 15, 2018 (day 79), to April 30, 2018 (30 days after the end of the NSC3 and Personal Pledge intervention period, day 184). Corporate Challenge participants were defined as NSC3 participants who enrolled in the Corporate Challenge. Non–Corporate Challenge participants were defined as those who did not enroll in the Corporate Challenge, either because they were not eligible or because they chose not to enroll.

### Outcome measures and covariates

#### Steps.

The primary outcome measure was step counts measured using the participants’ own tracker or a free NSC3 tracker. Days with negative counts were removed during the data cleaning process.

#### Demographic characteristics.

Demographic information (sex: male/female; age in years; height in centimeters; weight in kilograms) was self-reported via the NSC3 app. Age was collapsed into 6 categories: in years, 17–28, 29–38, 39–48, 49–58, 59–68, and >68. Body mass index (BMI) was calculated as weight (kg)/height (m)^2^, and classifications for Asian populations were followed (<18.5 underweight, 18.5–22.9 normal, 23.0–27.4 overweight, ≥27.5 obese) (21).

#### Day and weather covariates.

Day of the week and weather (daily recorded maximum temperature and total rainfall), from September 28, 2017, to April 30, 2018, were included as covariates as they potentially affect activity levels (22).

### Statistical methods

#### NSC3 main intervention.

Regression discontinuity design (RDD) was used to assess whether trends in step counts changed with the implementation of NSC3. Step counts were partitioned into the pre-intervention period, to establish a baseline (September 28, 2017, to October 27, 2017; days −30 to −1), and the intervention period (October 28, 2017, to March 31, 2018; days 0 to 154). RDD was used to estimate: 1) the trend in step counts during the pre-intervention period (pre-intervention slope), 2) the trend in step counts during the intervention period (intervention slope), and 3) the change in mean step counts from the pre-intervention to the intervention period (to give an estimate of the change in step counts attributed to NSC3). A fuzzy RDD model was used as participants could register anytime between days –30 and 154. Steps were fitted with a quadratic time trend (cubic trend did not improve the model) and adjusted for participants’ demographic characteristics, the day of the week, and weather. Participants were stratified based on sex and age group, and separate RDD models were fitted to each subgroup.

#### Personal Pledge.

The difference-in-difference (DID) method with fixed-effects regression was used to compare the change in step counts over time for participants who enrolled in the Personal Pledge (regardless of whether they completed their pledge, “Pledge enrollers”) compared with participants who did not enroll (“non–Pledge participants”), assuming the between-group difference would remain constant had there not been a Personal Pledge. To assess the plausibility of the common trends assumption required for DID, mean step counts by group and day during the pre-intervention period (September 28, 2017, to October 27, 2017; days −30 to −1) were visually inspected. The step counts were modeled as a function of day of the week, weather, a term indicating whether it was the intervention period (or the pre-intervention period), and a term estimating the differential change in step counts from before to during the Personal Pledge between the 2 groups. A multivariate logistic regression model was selected via Akaike information criterion (AIC) using participants’ sex, age group, and BMI group as covariates. The histograms of the propensity scores were plotted to ensure sufficient overlap between the distributions of the participants who enrolled and those who did not. The propensity scores were used to adjust for differences in characteristics between those who enrolled and those who did not, using inverse probability weighting for our DID estimator (23).

#### Corporate Challenge.

The DID model specified above was used to compare the change in step counts over time between participants who did and who did not enroll in the Corporate Challenge, although in this model, the step count partition was different; the pre-intervention (baseline) period was set from October 28, 2017, to January 14, 2018 (days 0–78), and the intervention period was set from January 15, 2018, to April 30, 2018 (days 79–184).

#### Sensitivity analyses.

To assess the robustness of findings, sensitivity analyses were performed. For participants with at least 1 day of valid step counts in both the pre-intervention and intervention periods (*n* = 149,220), missing step counts were imputed with individual mean steps during the pre-intervention and intervention periods, respectively. For the remaining participants (*n* = 262,308), missing step counts were imputed with individual mean steps.

The same procedure was repeated to perform sensitivity analysis on the Corporate Challenge. For participants with at least 1 day of valid step counts during the Corporate Challenge pre-intervention period (October 28, 2017, through January 14, 2018; day 0 to day 78) and at least 1 day of valid step counts during the Corporate Challenge intervention period (October 28, 2017, through April 30, 2018; day 0 to day 184; *n* = 226,536), missing step counts were imputed with the respective period’s individual mean steps. For the remaining participants (*n* = 113,383), missing step counts were imputed with individual mean steps.

To further assess the robustness of the findings, a further sensitivity analysis for the increase in steps due to NSC3 was performed on a subsample (due to computational constraints) of participants (*n* = 1,008) who had at least 3 and 14 days of valid step counts in the pre-intervention and intervention periods, respectively. Participants were randomly selected with stratification to represent the main subgroups in the participant pool (i.e., sex (male/female), BMI group (underweight, normal, overweight, obese), age group (in years, 17–28, 29–38, 39–48, 49–58, 59–68, >68)), giving a total of 48 subgroups (2 × 4 × 6); therefore 21 participants were randomly selected from each subgroup to obtain 1,008 participants (21 × 48). The models specified above were then fitted on the data set with and without the missing step count values imputed (See Web Appendix 2 and Web Appendix 3).

## RESULTS

### Participants and participant demographic characteristics

**Figure 1:**
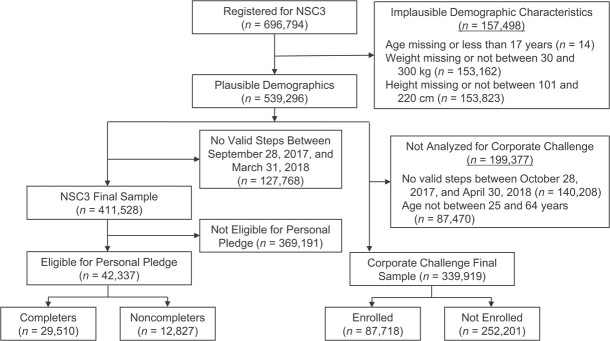
Flow diagram for data identification and access for National Steps Challenge Season 3 (NSC3), Personal Pledge, and Corporate Challenge, Singapore, 2017–2018. For NSC3 and Personal Pledge, the pre-intervention period was from September 28, 2017, to October 27, 2017 (days −30 to −1), and the intervention period was from October 28, 2017, to March 31, 2018 (days 0–154). *n* = 411,528 participants had at least 1 step count record between September 28, 2017, and March 31, 2018 (days −30 to 154). For Corporate Challenge, the pre-intervention period was from October 28, 2017, to January 14, 2018 (days 0–78), and the intervention period was from January 15, 2018, to April 30, 2018 (days 79–184). Following Organisation for Economic Co-operation and Development (48) classifications, participants were split into 3 groups: ages 15–24 years (those just entering the labor market following education), ages 25–54 (those in their prime working lives), and ages 55–64 (those passing the peak of their career and approaching retirement). *n* = 339,919 participants aged 25–64 had at least 1 step record between October 28, 2017, and April 30, 2018 (days 0–184).

In total, 696,794 people registered for NSC3. After excluding participants with missing or implausible demographic characteristics and without at least 1 valid day of step counts during the study period, *n* = 411,528 NSC3 participants were included in the present study (Figure 1). Of these, 10.3% (*n* = 42,337) and 21.3% (*n* = 87,718) enrolled in the Personal Pledge and the Corporate Challenge, respectively.

In NSC3, the mean age was 41.5 (standard deviation, 13.9) years, the mean BMI was 23.8 (standard deviation, 4.5), and there were more female (58.5%) participants (Table 1).

**Table 1:** Demographic Characteristics of the Sample for National Steps Challenge Season 3, Personal Pledge (Not Enrolled, Noncompleters, and Completers), and Corporate Challenge (Not Enrolled and Enrolled), Singapore, 2017–2018

**Sample**	**No. of Participants**	**No. of Observations**	**Mean (SD) Age, years**	**Mean (SD) BMI** ^**a**^	**Proportion Male, %**
NSC3^b^	411,528	30,642,140	41.5 (13.9)	23.8 (4.5)	41.5
Personal Pledge^b^^,^^c^					
Not enrolled	369,190	24,646,463	41.0 (13.8)	23.9 (4.6)	41.8
Enrolled					
Not completed	12,827	1,541,584	41.5 (12.6)	23.6 (4.1)	38.9
Completed	29,510	4,454,060	48.0 (14.1)	23.3 (3.9)	39.2
Corporate Challenge^c^^,^^d^					
Not enrolled	252,201	20,358,407	42.5 (10.8)	24.0 (4.4)	40.6
Enrolled	87,718	7,920,046	39.2 (9.6)	24.1 (4.6)	43.2

Abbreviations: BMI, body mass index; NSC3, National Steps Challenge Season 3; SD, standard deviation.

^a^ Weight (kg)/height (m)^2^.

^b^ NSC3 and Personal Pledge: pre-intervention period September 28, 2017, to October 27, 2017 (days −30 to −1); main intervention period October 28, 2017, to March 31, 2018 (days 0–154). Participants with at least 1 valid step record between September 28, 2017, and March 31, 2018 (days −30 to 154) are included.

^c^ One participant dropped because information on whether they took part in Personal Pledge and Corporate Challenge was not available.

^d^ Corporate Challenge: pre-intervention period October 28, 2017, to January 14, 2018 (days 0–78); intervention period January 15, 2018, to April 30, 2018 (days 79–184). Participants with at least 1 step record between October 28, 2017, and April 30, 2018 (days 0–184) are included. An additional participant was dropped as the registration date was after the start date of the Corporate Challenge.

### Distribution of propensity scores

For the propensity score models, the distribution of the propensity scores for participants who enrolled in the Personal Pledge (i.e., Pledge completers, Pledge noncompleters) was similar to participants who did not enroll in the Personal Pledge (i.e., non–Pledge participants) (Web Figure 1). The distribution of propensity scores for participants who did or did not enroll in the Corporate Challenge was also similar (Web Figure 1).

### Distribution of step counts

**Figure 2:**
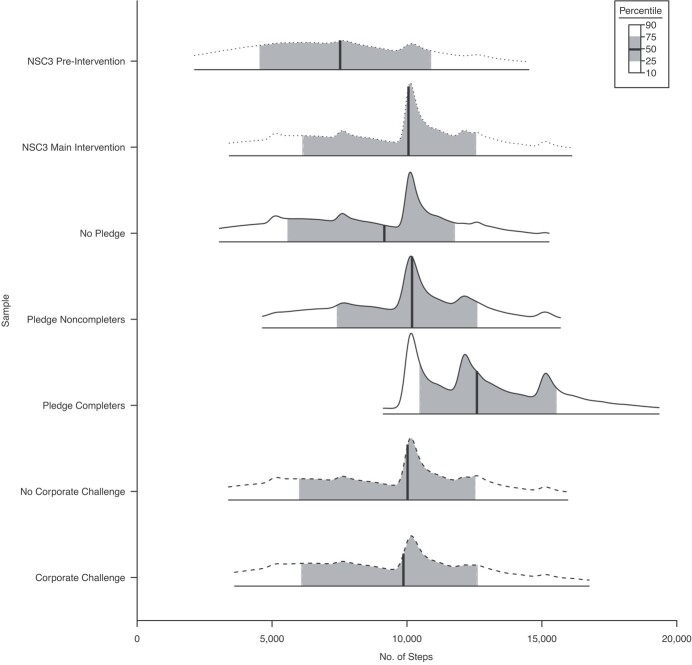
Distribution of daily step counts for National Steps Challenge Season 3 (NSC3) pre-intervention (*n* = 165,320), NSC3 main intervention (*n* = 395,428), Personal Pledge (no pledge (non–Pledge participants, *n* = 353,105), Pledge noncompleters (*n* = 12,812), Pledge completers (*n* = 29,510)), and Corporate Challenge (not enrolled (*n* = 196,344) and enrolled (*n* = 69,574)) during relevant intervention periods, Singapore, 2017–2018. The bars show the 10th, 25th, 50th (median), 75th, and 90th percentiles of the distribution, and 96.1% of NSC3 participants, 95.6% of non–Pledge participants, 99.9% of Pledge noncompleters, 100% of Pledge completers, 77.9% of non–Corporate Challenge participants, and 79.3% of Corporate Challenge participants had data during the intervention periods.

During the pre-intervention period (days −30 to −1), participants (*n* = 165,320) took a median of 7,523 steps per day (interquartile range (IQR), 4,555–10,877) compared with 10,056 steps per day (IQR, 6,141–12,546) during the NSC3 intervention period (days 0–154, *n* = 395,428) (Figure 2).

During the NSC3 intervention period (days 0–154), non–Pledge participants (*n* = 353,105) took a median of 9,161 steps (IQR, 5,590–11,758) while the Pledge noncompleters (*n* = 12,812) took a median of 10,189 steps (IQR, 7,419–12,591) and the Pledge completers (*n* = 29,510) took a median of 12,592 steps (IQR, 10,481–15,527). During the Corporate Challenge intervention period (days 79–184), non–Corporate Challenge participants (*n* = 196,344) took a median of 10,022 steps (IQR, 6,021–12,517), while Corporate Challenge participants (*n* = 69,574) took a median of 9,869 steps (IQR, 6,100–12,604). Although a total of *n* = 87,718 participants were enrolled in the Corporate Challenge, 79.3% of them had data during the intervention period. Similarly, 96.1% of NSC3 participants, 95.6% of non–Pledge participants, 99.9% of Pledge noncompleters, and 77.9% of non–Corporate Challenge participants had data during the intervention periods.

### NSC3 main intervention

**Figure 3:**
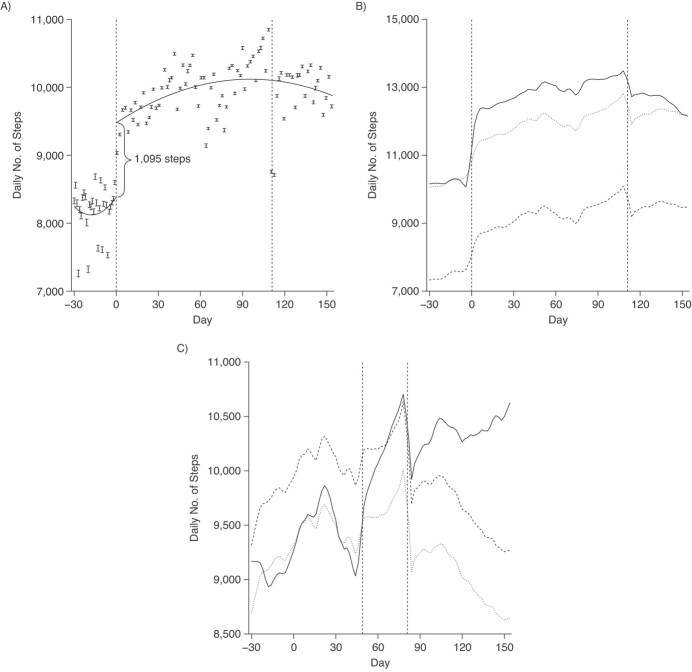
Steps trends in National Steps Challenge Season 3 (NSC3), Singapore, 2017–2018. A) Regression discontinuity design plot of daily step counts with quadratic time trends from September 28, 2017, to March 31, 2018 (days −30 to 154), *n* = 411,528. B) Daily steps mean trend of participants in Personal Pledge and not in Personal Pledge from September 28, 2017, to March 31, 2018 (days −30 to 154), *n* = 411,527. C) Daily steps mean trend of participants in Corporate Challenge and not in Corporate Challenge from October 28, 2017, to April 30, 2018 (days 0 to 184), *n* = 339,919. For (A), the observations are binned, and 95% nonparametric confidence intervals are computed (49). The solid boxes in the middle of the error bar represent the mean; the “whiskers” represent the 95% confidence interval. The solid line is the prediction based on the mean from the sharp regression discontinuity design model. For (B) and (C), the solid line is the mean trend of the participants who enrolled in the Personal Pledge (or Corporate Challenge). The dashed line is the mean trend of the participants who did not enroll in the Personal Pledge (or Corporate Challenge). The dotted line is the mean trend of the participants who did not enroll in the Personal Pledge (or Corporate Challenge) shifted downward by the difference between the two means at the start of Personal Pledge (Corporate Challenge). The increase in steps from Personal Pledge (or Corporate Challenge) is the difference between the solid line and the dotted line. One participant was dropped because information on whether they took part in the Personal Pledge and Corporate Challenge was not available. An additional participant was dropped for the Corporate Challenge as the registration date was after the start date. Key dates: NSC3 started on October 28, 2017, and ended on March 31, 2018 (days 0 to 154); Lunar New Year started on February 16, 2018, and ended on February 17, 2018 (days 111 to 112); Personal Pledge started on October 28, 2017, and ended on March 31, 2018 (days 0 to 154); Corporate Challenge started on January 15, 2018, and ended on April 30, 2018 (days 79 to 184).

**Figure 4:**
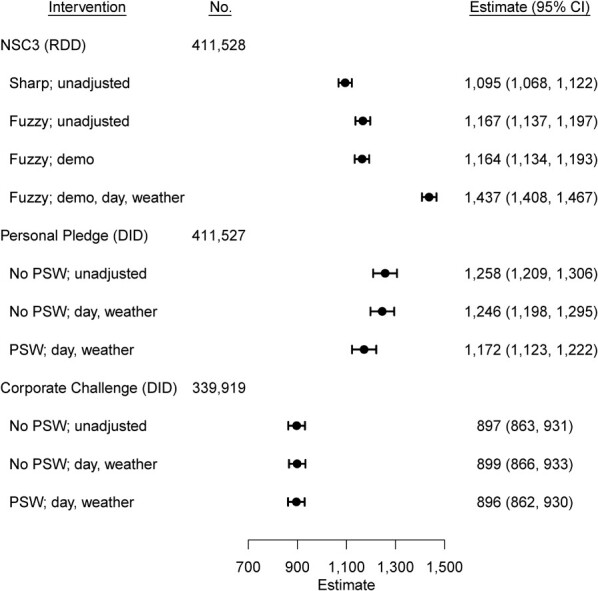
Estimated steps increase using regression discontinuity design (RDD) of National Steps Challenge Season 3 (NSC3) daily step counts from September 28, 2017, to March 31, 2018 (days −30 to 154), difference-in-difference (DID) with propensity score–weighted (PSW) fixed-effects regression of Personal Pledge daily step counts from September 28, 2017, to March 31, 2018 (days −30 to 154), and difference-in-difference with propensity score weighted fixed-effects regression of Corporate Challenge daily step counts from October 28, 2017, to April 30, 2018 (days 0 to 184), adjusting for different covariates. The covariates are demographic characteristics (demo: age group, sex, body mass index group); day (day of the week); weather (maximum temperature, log (1 + rainfall)). 95% confidence intervals (CIs) were constructed using standard errors clustered at the participant level. Sharp RDD assumes that every participant signed up on the start date of NSC3, October 28, 2017 (day 0). In contrast, fuzzy RDD relaxes this assumption by allowing participants to register at any time during the NSC3 intervention period. Fixed-effects regression controls for all time-invariant variables, such as demographic characteristics. One participant was dropped because information on whether they took part in Personal Pledge and Corporate Challenge was not available. An additional participant was dropped for the Corporate Challenge as the registration date was after the start date.

After the start of NSC3, there was an associated increase in the step counts by 1,095 steps (95% confidence interval (CI): 1,068, 1,122) per day (Figure 3A). Using the fuzzy RDD model and adjusting for covariates, NSC3 was associated with a mean increase of 1,437 steps (95% CI: 1,408, 1,467) per day (Figure 4). For NSC3, the associated mean increase varied by age and sex groups (Web Figures 2 and 3). Younger age groups (17–28 and 29–38 years) had lower pre-intervention step counts and lower increases in step counts than older age groups. Across all age groups, female participants had a greater increase in step counts compared with male participants.

### Personal Pledge

Of the 42,337 NSC3 participants who enrolled in the Personal Pledge, 69.7% (*n* = 29,510) completed their pledge (Table 1). Step counts for both completers and noncompleters increased steadily until day 111 (Lunar New Year) (Figure 3B).

During the pre-intervention period (days −30 to −1), completers and noncompleters (i.e., Pledge enrollees, *n* = 42,337) were taking an average of 2,446 steps (95% CI: 2,389 to 2,974) per day more than non–Pledge participants (*n* = 369,190; of whom 12.9% (*n* = 47,618) were eligible). During the intervention period (days 0–154), after adjusting for the day of the week and weather and weighting using the propensity scores, enrolling in the Personal Pledge was associated with an additional mean increase of 1,172 steps (95% CI: 1,123, 1,222) per day (Figure 4).

### Corporate Challenge

Of the 82.6% (*n* = 339,919) of NSC3 participants eligible for Corporate Challenge, 25.8% (*n* = 87,718) enrolled and 74.2% (*n* = 252,201) did not (Table 1).

During the pre-intervention period (days 0–78), mean step count trends were similar among participants who subsequently enrolled or did not enroll in the Corporate Challenge (Figure 3C). During the Corporate Challenge intervention period (days 79–184), after adjusting for the day of the week and weather and further weighting the observations using the propensity scores, enrolling in the Corporate Challenge was associated with an additional mean increase of 896 steps (95% CI: 862, 930) per day (Figure 4).

While both Corporate Challenge participants and non–Corporate Challenge participants experienced a drop in mean steps during the Lunar New Year (day 111–112), the mean steps of Corporate Challenge participants increased to pre–Lunar New Year levels by the end of the Corporate Challenge period (day 184).

### Sensitivity analyses

Following the imputation of missing step counts using the participant’s mean steps, NSC3 was associated with a mean increase of 1,135 steps (95% CI: 1,120, 1,150) per day. Enrolling in the Personal Pledge was associated with an additional mean increase of 2,071 steps (95% CI: 2,028, 2,113) per day. Enrolling in the Corporate Challenge was associated with an additional mean increase of 682 steps (95% CI: 659, 704) per day (Web Figure 4).

Following imputation of missing step counts using the Bayesian hierarchical model, NSC3 was associated with a mean increase of 1,336 steps (95% CI, 988 to 1,683) per day (Web Figure 5).

## DISCUSSION

### Main findings

We examined the impact of NSC3, a scaled-up mHealth intervention promoting physical activity in Singapore. Overall, there was an associated mean increase of 1,437 steps per day following the start of the NSC3. This change was larger among participants with higher step counts during the pre-intervention period and among female and older participants. The nested booster challenges, Personal Pledge and Corporate Challenge, were associated with additional mean increases of step counts by 1,172 and 896 steps per day, respectively. All 3 associated step counts increases were sustained for at least 1 month from the start of the intervention period.

The associated increase of 1,437 steps per day compares favorably with other physical activity interventions. Meta-analyses (6, 24, 25) of smartphone and mHealth interventions report increases of 477–1,850 steps per day under controlled experimental conditions, where intervention effects are known to be stronger (26). Currently, there are few studies reaching the scale and real-world implementation approach of NSC3 to draw comparisons with. One recent review of scaled-up interventions observed effect sizes ranging from 29%–75% (median, 66%) of the associated pre–scale-up efficacy trial (27). Meta-analysis estimating the impact of Pokémon GO (28) with a pooled sample size of *n* = 33,108 observed similar changes in step counts compared with NSC3. That NSC3 compares favorably is promising given the relative simplicity of the intervention, with its clear focus on physical activity, and the sheer scale of the intervention. NSC has reached over 1.3 million unique participants (over 1/3 of Singapore's population has joined) (17, 29).

Our results demonstrate the potential benefits of embedding additional booster challenges within a larger intervention. Enrolling in the Personal Pledge was associated with a mean increase of 1,172 steps per day. This increase may partially reflect inherent differences in these participants (compared with the whole NSC3 sample) or may be driven by their established commitment to NSC along with the additional goal-setting these participants were engaged in. Once Personal Pledge participants reached the final stage of NSC, they were able to select their own step count goal. The personalized goal setting and additional incentives associated with Personal Pledge may have provided extrinsic motivation, acting as strategies to draw these participants back to the intervention (30–33).

Corporate Challenge commenced almost 3 months into NSC3 and was associated with a mean increase of 896 steps per day. This contrasts with the broader pool of NSC3 participants whose step counts had started to decline by that time, which is similar to other physical activity interventions (24, 34). The team-based structure of Corporate Challenge may have encouraged positive peer effects (35, 36) and collective empowerment. Employees in the same team (company) may motivate one another to increase their step counts to obtain both individual and collective incentives (37, 38), leading to a larger increase in average step counts. Meta-analyses indicate that financial incentives are an effective intervention component (37). The combination of individual and collective incentives may be particularly effective (38) and may be more cost-effective than larger individual incentives. To maximize the impact of a physical activity intervention stretched across 5 months, implementing a booster initiative every 90 days (when intervention effects typically seem to dissipate) may be helpful. We observed that participants tended to regress to their physical activity from before the pledge levels after the Personal Pledge and upon the completion of their pledge goals. Therefore, it is important to calibrate the pledge goal and pledge duration to encourage habit formation that would lead to the long-term persistence of sufficient physical activity. More research is needed to determine the ideal time frame for these boosters.

In our study, small incentives were tied to achieving step count targets lower than what would equate to guideline amounts (3), and we still saw considerable increases in physical activity. This has important implications for population-level interventions as incentives could be tied to individuals’ starting point or ability which may reduce barriers to joining an intervention. Importantly, even small increases in step counts have health benefits, such as reducing the incidence of hypertension, improving cardiovascular health biomarkers, and reducing BMI (39). In Singapore, the economic cost of physical inactivity is estimated to be equivalent to the purchasing power of US $201 million in direct costs (for diseases directly attributable to physical inactivity such as heart diseases, type 2 diabetes, some cancers etc.) and indirect costs (productivity loss due to morbidity and premature mortality) (40). Small financial incentives to individuals for engaging in physical activity may therefore be a cost-effective strategy to reduce direct and indirect costs of physical inactivity. While the approach is promising, NSC3 is unique in its scale and reach (41), and further research is needed to determine whether our results are replicable.

Limitations of the approach should be noted. Being smartphone-based may explain the underrepresentation of older age groups, who may be less tech-savvy and therefore less inclined to join the intervention (compared with younger users). Nevertheless, with no program signup fees, the NSC is accessible to all individuals regardless of socioeconomic status*.* Another limitation is the cost of conducting NSC, which could be spent advancing other (possibly nonhealth) goals that policy makers may have. Future research could investigate the cost-effectiveness of NSC.

Limitations of the approach should be noted. Residual confounding is still possible. However, to encourage enrollment into NSC, participant demographic and other factors that could potentially bias the results (e.g., distance to nearest park) were not collected. If available, this information could allow for an in-depth evaluation of NSC from an implementation perspective, using an appropriate framework (45, 46). The generalizability of our findings may be limited by selection bias, because participants self-selected into the study. Further, social desirability biases may have influenced missing days of step data (47); participants may have been less likely to upload their data on days when they took fewer steps. To mitigate this limitation, we performed extensive sensitivity analyses and observed that the results were robust and consistent with the primary analyses (see Web Appendix 4, Web Figures 6–9). Also, although our sample is large, participants are not representative of the overall population of Singapore. We observed that women and people with overweight or obesity (compared with those with normal weight) were more likely to join NSC than population norms would suggest. The Corporate Challenge participants were overrepresented for women, in the younger age group, and in the education, public, and manufacturing sectors (see Web Appendix 5, Web Figures 10 and 11). Being smartphone-based may explain the underrepresentation of older age groups, who may be less tech-savvy and therefore less inclined to join the intervention (compared with younger users). Nevertheless, with no program signup fees, the NSC is accessible to all individuals regardless of socioeconomic status. Another limitation is the cost of conducting the NSC, which could be spent advancing other (possibly nonhealth) goals that policy makers may have.

A key strength of our study is the quasi-experimental design. Additionally, although residual confounding is possible, we further reduced the potential for it by adjusting for demographic (i.e., age, sex, BMI) and environmental factors (i.e., weather (43, 44)) that are known to have an impact on the performance of physical activity, which may be of particular importance in Singapore's year-round hot and humid climate (22). Further strengths are our large sample of over 411,000 individuals who collectively recorded over 30 million steps in a real-world intervention setting over 5 months. To the best of our knowledge, no other physical activity intervention has reached the scale and reach of NSC3.

A key implication of this study is the possibility of conducting an effective nationwide physical activity intervention. With more than 500 million people estimated to have downloaded an mHealth application on their smartphone globally (42), policy makers could leverage this technology to deliver effective large-scale physical activity interventions. Future research could investigate the cost-effectiveness of the NSC, as well as exploring issues of equitable access and equitable benefits to health in the context of the NSC (e.g., enrollment of inactive people who stand to gain the most).

### Conclusion

Participants in Singapore’s National Steps Challenge Season 3 saw substantial increases in their step counts during the 5-month intervention period and during the 2 booster challenges, Personal Pledge and Corporate Challenge. Findings suggest that mHealth interventions with small incentives and peer effects are an effective strategy to increase population-wide physical activity levels.

## Supplementary Material

Web_Material_kwac193
