# Supplementary material for: Physical Activity Trends Among Adults in a National Mobile Health Program: A Population-Based Cohort Study of 411,528 Adults
Source: Am J Epidemiol. 2022 Nov 7;192(3):397–407. doi: 10.1093/aje/kwac193 (PMC9985108; doi:10.1093/aje/kwac193)
Supplement: Web_Material_kwac193 [file web_material_kwac193.pdf]

WEB MATERIAL

Physical Activity Trends Among Adults in a National mHealth Program: A Population-Based Cohort Study of 411,528 Adults

Gregory Ang, Sarah Martine Edney, Chuen Seng Tan, Nicole Lim, Jeremy Tan, Falk Müller-Riemenschneider, Cynthia Chen

Web Appendix 1. Information on National Steps Challenge, Personal Pledge and Corporate Challenge.

Web Appendix 2. Sensitivity Analyses.

Web Appendix 3. Mathematical Appendix.

Web Appendix 4. Comparison of Corporate Challenge participants to the general population.

Web Appendix 5. Treating age and body mass index as continuous variables.

Web Table 1. Earning Health Points in the NSC3 rewards tier system.

Web Table 2. Exchanging Health Points for sure-win vouchers.

Web Table 3. Possible combinations of days and steps for Personal Pledge.

Web Figure 1. Propensity score distribution and overlap of participation in Personal Pledge and Corporate Challenge.

Web Figure 2. Daily Steps Mean trend of NSC3 sample from 28 September 2017 to 30 April 2018 (days -30 to 184), by gender and age groups.

Web Figure 3. Estimated steps increase using regression discontinuity design of NSC3 from 28 September 2017 to 31 March 2018 (days -30 to 154), by gender and age groups.

Web Figure 4. Sensitivity analysis 1: Estimated steps increase using regression discontinuity design of NSC3 daily step counts from 28 September 2017 to 31 March 2018 (days -30 to 154), difference-in-difference with fixed-effects regression of Personal Pledge daily step counts from 28 September 2017 to 31 March 2018 (days -30 to 154) and difference-in-difference with fixed-effects regression of Corporate Challenge daily step counts from 28 October 2017 to 30 April 2018 (days 0 to 184), adjusting for different covariates; with and without imputation.

Web Figure 5. Sensitivity analysis 2: Estimated steps increase using regression discontinuity design of NSC3 from 28 September 2017 to 31 March 2018 (days -30 to 154) with and without mean imputation.

Web Figure 6. Comparison of industries between Corporate Challenge participants and employed Singapore residents aged between 25 to 64 years.

Web Figure 7. Comparison of sex and age group between Corporate Challenge participants and employed Singapore residents aged 25 to 64 years.

Web Figure 8. Sensitivity analysis 1: NSC3 regression discontinuity design plot of daily step counts with quadratic time trends from 28 September 2017 to 31 March 2018 (days -30 to 154), n = 411,528.

Web Figure 9. Sensitivity analysis 1: Daily Steps Mean trend of those in Personal Pledge and not in Personal Pledge from 28 September 2017 to 31 March 2018 (days -30 to 154), n = 411,527.

Web Figure 10. Sensitivity analysis 1: Daily Steps Mean trend of those in Corporate Challenge and not in Corporate Challenge from 28 October 2017 to 30 April 2018 (days 0 to 184), n = 339,919.

Web Figure 11. Comparison of estimated increase using regression discontinuity design using different covariates and interrupted time series of NSC3 step counts from 28 September 2017 to 31 March 2018 (days -30 to 154) (n = 411,528).

Web Appendix 1. Information on National Steps Challenge, Personal Pledge and Corporate Challenge.

This section elaborates on the incentive structure of the National Steps Challenge Season 3 (NSC3), as well as the Personal Pledge and Corporate Challenge. All information on the overall National Steps Challenge intervention and Personal Pledge was obtained from the National Steps Challenge Season 3: Frequently Asked Questions (1). All information on the Corporate Challenge was obtained from Corporate Challenge Frequently Asked Questions (FAQs) (2).

Rewards structure

Participants could earn Health Points on each day of NSC3, as shown in **Web Table 1.** Health Points can be exchanged for vouchers for various retail outlets (e.g., supermarkets, sports retailers). This tiered system of daily step targets was intended to incentivize even small increases in physical activity. Rewards were gain-framed (i.e., "sure-win") to ensure as few barriers to joining the intervention as possible. (3)

Web Table 1. Earning Health Points in the NSC3 rewards tier system.

Steps per day	Health Points (HP)
5,000	10
7,500	25
10,000	40

Web Table 2. Exchanging Health Points for sure-win vouchers.

Tier	Health Points (HP)	Sure-Win Reward (voucher)
1	750	$5
2	Additional 1,500	$10
3	Additional 750	$5
4	Additional 750	$5
5	Additional 750	$5
6	Additional 750	$5

Overall NSC3 intervention

NSC3's intervention period was from 28 October 2017 to 31 March 2018 (days 0 to 154). During this 155-day period, participants used their own step tracker (i.e., commercial wearable, smartphone accelerometer) or a free NSC3 step tracker (most commonly used trackers were HPB Careeach (21.5%) and inbuilt smartphone accelerometers (Apple Healthkit 15.7%, Samsung 13.6%)), provided as part of the intervention, to monitor their daily step counts and participants were periodically reminded to sync their tracker with the NSC3 app to record their step counts over time. Points were awarded for reaching certain step count thresholds (e.g., achieving 5,000, 7,500 or 10,000 steps in a day earnt 10, 25 or 40 points, respectively), and these could be exchanged for cash vouchers. **Web Table 2** shows the amount of HP required to obtain the sure-win vouchers. Points also unlocked new challenge tiers; participants start on Tier 1 and need to have accumulated 5250 points to reach Tier 6 (final tier). Every 750 points earned participants $5, therefore participants earned $35 upon the completion of all 6 tiers. Individuals who had participated in NSC seasons 1 and/or 2 continued from the last tier they unlocked.

Personal Pledge

For participants that have completed all six tiers, they are then able to take part in the optional Personal Pledge. Participants selected their own step count goal and the number of days they aimed to achieve it (e.g., 10,000 steps for 90 days, 15,000 steps for 30 days; days need not be consecutive). The participant can pledge in one category, which is chosen at the start and once chosen, cannot be changed. The

categories are presented in **Web Table 3**. Personal Pledge ran for the entirety of the main intervention period (i.e., 28 October 2017 to 31 March 2018, days 0 to 154) and participants had from the day of their pledge or from the beginning of the main intervention period (whichever was later) until 31 March 2018 to fulfil their pledge and be eligible for rewards of up to $70 (1).

Web Table 3. Possible combinations of days and steps for Personal Pledge.

Days/Steps per day	10,000	12,000	15,000
**15**			✓
**30**		✓	✓
**60**	✓	✓	✓
**90**	✓	✓	✓
**120**	✓	✓	✓

Corporate Challenge

The Corporate Challenge period was conducted from 15 January 2018 (79 days after the start of the NSC3 intervention period) to 30 April 2018 (30 days after the end of the NSC3 and Personal Pledge intervention period, day 184). Based on the Organisation for Economic Co-operation and Development (OECD), they classified employment rates into three age groups: people aged 15 to 24 (those just entering the labour market following education); people aged 25 to 54 (those in their prime working lives); people aged 55 to 64 (those passing the peak of their career and approaching retirement). Hence, we only kept observations from individuals aged 25 to 64.

There were two Corporate Challenge categories: organisations with more than or with less than 200 employees. For each category, the company with the three highest daily average step counts per participant received rewards (e.g., first prize of $10,000 and $5,000 for organisations with >200 and <200 employees, respectively). Also, there were monthly prizes available.

Results

For the Personal Pledge and Corporate Challenge, the histograms of the propensity scores were plotted (**Web Figure 1**). There was sufficient overlap between the distributions of the participants who enrolled and those who did not. For NSC3, the associated mean increase varied by age and sex groups (**Web Figure 2, Web Figure 3**). Younger age groups (17-28 and 29-38 years) had lower pre-intervention step counts and lower increases in step counts than older age groups. Across all age groups, females had a greater increase in step counts compared to males.

Web Appendix 2. Sensitivity Analyses.

This section describes the sensitivity analysis done to investigate the robustness of the estimates of the increase in steps due to NSC3, Personal Pledge, and the Corporate Challenge. The goal of this analysis is to illustrate that the estimates are similar with and without imputing the missing step counts.

We conducted two different sensitivity analyses described in more detail below. The results of the sensitivity analyses are presented in **Web Figure 4** and **Web Figure 5**.

(I) Sensitivity analysis 1: using the participant's mean step counts

Sample Selection

Main NSC3 intervention and Personal Pledge

Based on our NSC3 sample of 411,528 participants, there were 149,220 participants with at least one observation during the pre-intervention period (28 September 2017 – 27 October 2017; day -30 to day -1) and at least one observation during the intervention period (28 October 2017 – 31 March 2018; day 0 to day 154). For each of these 149,220 participants, we computed the mean step counts before and during the interventions. Using that participant's mean, we then imputed the participant's missing observation, depending on whether that time point was before or during the intervention.

For the remaining 262,308 participants, the participant's missing step counts were imputed using their mean steps computed from the observed data. Hence for these 262,308 participants, we assumed there was no change in steps due to NSC3 or the Personal Pledge.

Corporate Challenge

Similarly for the Corporate Challenge sample of 339,919 participants, there were 226,536 participants with at least one observation during the Corporate Challenge pre-intervention period (28 October 2017 – 14 Jan 2018; day 0 to day 78) and at least one observation during the Corporate Challenge intervention period (28 October 2017 – 30 April 2018; day 0 to day 184). For each of these 226,536 participants, we then imputed the respective periods' mean step counts.

For the remaining 113,383 participants, the participant's missing step counts were imputed using their mean steps computed from the observed data. Hence for these 113,383 participants, we assumed there was no change in steps due to the Corporate Challenge.

Model Specification

Regression discontinuity design and difference-in-difference with fixed-effects regression models were then used to estimate the increase in steps based on two datasets: (i) the observed step counts without imputation and (ii) the observed step counts with imputation.

Results

By imputing the missing observations with the participant's mean, we observed that the daily mean step counts fell for all groups, with the smallest associated decrease for the participants who enrolled in the Personal Pledge. After the start of NSC3, there was an associated increase in the step counts by 571 steps (95% Confidence Interval (CI), 562 to 580) per day (**Web Figure 8**). Using the fuzzy RDD model and adjusting for covariates, NSC3 was associated with a mean increase of 1135 steps (95% CI, 1120 to 1150) per day (**Web Figure 4**). After adjusting for day of the week and weather, and weighting using the propensity scores, enrolling in the Personal Pledge was associated with an additional mean increase of 2071 steps (95% CI, 2028 to 2113) per day (**Web Figure 4, Web Figure 9**), while enrolling in the Corporate Challenge was associated with an additional mean increase of 682 steps (95% CI, 659 to 704) per day (**Web Figure 4, Web Figure 10**).

(II) Sensitivity analysis 2 (using a model-based approach)

This section describes the further sensitivity analysis done to investigate the robustness of the estimate of the increase in steps due to the National Steps Challenge Season 3 (NSC3). We built a Bayesian Hierarchical Model, where the model parameters were estimated using Markov Chain Monte Carlo (MCMC).

Sample Selection

Based on our NSC3 sample of 411,528 participants, we further removed participants by only retaining individuals with at least three days of observations during the pre-intervention period and at least two weeks of observations during the intervention period. These cut-offs were chosen as the pre-intervention period, and the intervention period was approximately 4 and 20 weeks, respectively. Madley-Dowd et al. (2019) (4) showed via using simulations that unbiased results could be obtained even with 90% of missing data, provided the imputation model was properly specified, and data were missing at random.

Based on the cut-offs, we ended with 116,430 participants. We had two genders (male/female), four BMI groups ((i) Normal: 18.5 to 23, (ii) Underweight: less than 18.5,(iii) Overweight: 23 to 27.5 and (iv) Obese: greater 27.5) and six age groups (17 to 28, 29 to 38, 39 to 48, 49 to 58, 59 to 68, greater than 68); making a total of 48 subgroups (2 x 4 x 6). From the 116,430 participants, we randomly selected 21 participants from each subgroup to obtain 1008 participants (21 x 48).

The first filtering step was done as a participant level model will be built, and sufficient data points are needed to estimate the trends. As an extreme example, we need at least two data points to estimate a trend. The second filtering step was done due to computational constraints; for 1008 participants, we needed enough computer memory to store data for approximately 186 thousand person-days for 40,000 samples for over 40 parameters.

Model Specification

A participant level longitudinal model was built to model the step counts throughout the entire NSC3 intervention period. The model was hierarchical to account for the differences between participants and accounted for the time trend of the step counts during the NSC3 intervention period. The step counts at any time point was positive and modelled via a Gaussian-distributed variable, $\mathrm{Steps}_{it} \sim \mathrm{N}(\mu_{it}, \sigma^2)$, for participant *i* at time *t*. The mean step counts, μ_{it}, varies by individual, time, age group, gender, BMI group, day of the week, maximum temperature and log(1+rainfall). We assume a constant time trend before the NSC3 intervention and a quadratic time trend during the intervention. The mean step counts, μ_{it}, for participant *i* at time *t* can, therefore, be expressed as

$$\mu_{it} = \beta_{0,i} + \beta_{1,i} I(t \geq 0) + \beta_{2,i} I(t \geq 0)t + \beta_{3,i} I(t \geq 0)t^2 + \beta_{4,i} MaxTemperature_t + \beta_{5,i} \log(1 + Rainfall_t)$$

$$+ \sum_{j=2}^{7} \beta_{4+j,i} I(DayofWeek_t = j) + \sum_{j=2}^{4} \beta_{10+j,t} I(BMIgroup_i = j) + \sum_{j=2}^{6} \beta_{13+j,t} I(Agegroup_i = j) + \beta_{20,t} I(Male)$$

We chose non-informative priors. Location parameters were given Gaussian priors while scale parameters were given Inverse Gamma priors. The estimation was implemented in RStan (5). We ran four independent chains with 10,000 iterations. The first 5000 runs for each chain were discarded as burn-in. Point estimates are posterior mean values, and uncertainty intervals are 95% credible intervals.

$$\beta_{j,i} \sim N(\gamma_j, \tau_j^2) \quad j \in \{0,1,\dots,11\},$$

$$\beta_{j,t} \sim N(\gamma_j, \tau_j^2) \quad j \in \{12,\dots,20\},$$

$$\gamma_j \sim N(0, 100^2) \quad j \in \{0,1,\dots,20\},$$

$$\tau_j \sim \text{InvGamma}(0.001,0.001) \quad j \in \{0,1,\dots,20\},$$

$$\sigma \sim \text{InvGamma}(0.001,0.001).$$

Results

Using the Bayesian Hierarchical model, we used the individual's predicted mean to impute the missing step counts. After that, we reran the fuzzy RDD model on two datasets: (i) The observed step counts of the 1008 individuals only and (ii) dataset (i) with the missing values imputed with the posterior mean. Based on datasets (i) and (ii), NSC3 was associated with a mean increase of 1449 steps (95% CI 1127 to 1771) per day and 1518 steps (95% CI 1228 to 1809) per day, respectively (**Web Figure 5**). Also, we reweighted the observations based on their demographic proportions in their NSC3 sample. Upon reweighting, based on datasets (i) and (ii), NSC3 was associated with a mean increase of 1276 steps (95% CI 887 to 1665) per day and 1336 steps (95% CI 988 to 1683) per day, respectively.

Web Appendix 3. Mathematical Appendix.

We provide a mathematical explanation to the models used to estimate the increase in steps in this section.

(I) National Steps Challenge Season 3 (NSC3)

Selection of quadratic time trend

Firstly, we grouped the observations and computed non-parametric confidence intervals (8). We fitted regression models and superimposed the predicted mean curve for $p \in \{1,2,3\}$.:

$$Steps_{it} = \beta_0 + \beta_1 T_{it} \sum_{j=1}^{p} \left[\beta_{2j} t_i^j + \beta_{2j+1} T_{it} t_i^j\right] + \epsilon_{it}$$

where

- $Steps_{it}$ are the step counts of participant i at time t. Step counts were fitted with a quadratic time trend as a cubic trend did not improve the model.
- T_t is the indicator variable for the intervention period and equals one if t is greater than zero, zero otherwise.

Sharp Regression Discontinuity Design

We selected $p = 2$. We fit the following regression model

$$Steps_{it} = \beta_0 + \delta T_{it} + \beta_1 t_i + \beta_2 T_{it} t_i + \beta_3 {t_i}^2 + \beta_4 T_{it} {t_i}^2 + \boldsymbol{\theta}\, \boldsymbol{X_{it}} + \varepsilon_{it},$$

where

- $Steps_{it}$ are the step counts of participant i at time t. Step counts were fitted with a quadratic time trend as a cubic trend did not improve the model.
- T_t is the indicator variable for the intervention period and equals one if t is greater than zero, zero otherwise.
- $\boldsymbol{X_{it}}$ are the participants' demographics, day of the week and weather.

Fuzzy Regression Discontinuity Design

The Sharp RDD assumes that all participants with step counts from 28 Oct 2017 to 31 Mar 2018 were eligible for the NSC3 rewards. However, participants also needed to register before they are eligible. To account for the individuals who registered after 28 Oct 2017, the increase in steps was estimated via the two-stage procedure described below:

First Stage

We regressed the treatment variable D_{it} on t, t^2, the cutoff variable T_t and their interaction terms.

$$D_{it} = \alpha_0 + \alpha_1 T_t + \alpha_2 t + \alpha_3 T_t t + \alpha_4 t^2 + \alpha_5 T_t t^2 + \boldsymbol{\alpha Z_{it}^{(1)}} + \nu_{it}. \quad (1)$$

We also constructed analogous first stages by replacing the dependent variable D_{it} with $D_{it}t$ and $D_{it}t^2$.

Second Stage

Using the predicted values obtained from the first stage ($\widehat{D_{it}}$, $\widehat{D_{it}t}$, $\widehat{D_{it}t^2}$), we conducted the second stage regression.

$$Steps_{it} = \beta_0 + \beta_1 \widehat{D_{it}} + \beta_2 t + \beta_3 \widehat{D_{it}t} + \beta_4 t^2 + \beta_5 \widehat{D_{it}t^2} + \boldsymbol{\beta Z_{it}^{(1)}} + \epsilon_{it}, \quad (2)$$

where

- $Steps_{it}$ are the step counts of participant i at time t. Step counts were fitted with a quadratic time trend as a cubic trend did not improve the model.

- D_{it} is the indicator variable for NSC3 and equals one if participant i is eligible for the incentive on day t, zero otherwise. The participant is only eligible for the incentive if the step count is done during the intervention period and the participant has registered before day t.
- T_t is the indicator variable for the intervention period and equals one if t is greater than zero, zero otherwise.
- $\boldsymbol{Z}_{it}^{(1)}$ are the participants' demographics, day of the week and weather.

(II) Personal Pledge and Corporate Challenge

Difference-in-difference (DID) method with fixed-effects regression was used to compare the change in step counts over time for participants:

(i) who enrolled in the Personal Pledge (regardless of whether they completed their pledge, "Pledge Enrollers") against participants who did not enrol ("non-pledge participants"). ; The pre-intervention (baseline) period was set from 28 September 2017 to 27 October 2017 (days *t* = -30 to -1), and the intervention period was set from 28 October 2017 to 31 March 2018 (days *t* = 0 to 154);

(ii) who enrolled and who did not enrol in the Corporate Challenge. The pre-intervention (baseline) period was set from 28 October 2017 to 14 January 2018 (days *t* = 0 to 78) and the intervention period was set from 15 January to 30 April 2018 (days *t* = 79 to 184).

The DID model assumed the between-group difference would remain constant had there not been a Personal Pledge (or Corporate Challenge). The model is of the form:

$$Steps_{it} = \beta_{0,i} + \beta_1 T_t + \beta_2 G_i \times T_t + \boldsymbol{\beta} \boldsymbol{Z}_t^{(2)} + \epsilon_{it}, \quad (3)$$

where

- $Steps_{it}$ are the step counts of participant i at day t.
- T_t is the indicator variable for the intervention period and equals one if day t is greater than zero, zero otherwise.
- G_i is the group variable and equals one if participant enrolled in the Personal Pledge (or Corporate Challenge).
- $\boldsymbol{Z}_t^{(2)}$ are the day of the week and weather.

We assessed the plausibility of the common trends assumption required for DID by visually inspecting the group trends of mean step counts during the pre-intervention period (28 September 2017 to 27 October 2017, days *t* = -30 to -1).

A multivariable logistic regression model was selected via Akaike Information Criterion (AIC) using the group variable (G_i) as the response variable and the participant's sex, age group, and BMI group as covariates. The predicted probabilities (p_i) were used to adjust for differences in characteristics between those who enrolled and those who did not, using inverse probability weighting for our DID estimator (9). The weights (w_i) were one if participant i enrolled in the intervention, $\frac{p_i}{1-p_i}$ otherwise. A weighted regression using equation (3) with weights (w_i) was performed.

Web Appendix 4. Comparison of Corporate Challenge participants to the general population.

For the 87,718 participants who enrolled in the Corporate Challenge, we had a variable "type of industry" that participants' jobs were classified as. This data was compared against data on employed residents in Singapore, obtained from the 2018 census (7).

Industry

We provide a comparison between the industries that Corporate Challenge participants are employed in, as compared to the industries of all employed residents in Singapore (aged between 25 to 64 years, inclusive).

Web Figure 6 shows the industries that Corporate Challenge participants were employed in. There was a high percentage of Corporate Challenge participants from the 'Education and Public Sector' (27.8%) as compared to the population data (11.9%). This over-representation is likely explained by strong employer-led support for the intervention, as Corporate Challenge (and National Steps Challenge) are initiatives of the public sector Health Promotion Board. There was also a high percentage and overrepresentation of Corporate Challenge participants (19.6%) in the 'Manufacturing' sector compared to the census (11.1%). There was an underrepresentation of participants in the Corporate Challenge from the 'Business Services' sector (5.7%) and from the 'Wholesales & Retail Trade' sector (4.1%).

Demographics

We also compared the demographics of the Corporate Challenge participants against employed residents in Singapore (aged between 25 to 64 years, inclusive)**.**

There were more female participants (CC: 56.8% vs Census: 45.7%) and participants tend to be younger compared to the general population of employed residents (**Web Figure 7**). 71.5% of Corporate Challenge participants aged between 25 to 64 were below age 45 while 52.5% of employed residents aged between 25 to 64 were below age 45. One possible reason for the underrepresentation of older age groups is that the National Steps Challenge is an app-based intervention, which may appeal more to younger users.

Web Appendix 5. Treating age and body mass index as continuous variables.

During the analysis, we categorized age and body mass index (BMI) based on groups. Age was collapsed into six categories: 17-28, 29-38, 39-48, 49-58, 59-68, and >68 years. Body mass index (BMI) classifications for Asian populations were followed (<18.5 underweight, 18.5-22.9 normal, 23-27.4 overweight, >27.5 obese) (6).

We further re-ran the regressions using three different specifications (i) age groups and BMI groups, (ii) age and BMI as continuous variables, and (iii) age, age^2, BMI, and BMI^2 as continuous variables (**Web Figure 11**). From the results, the estimates do not differ much based on the different specifications.

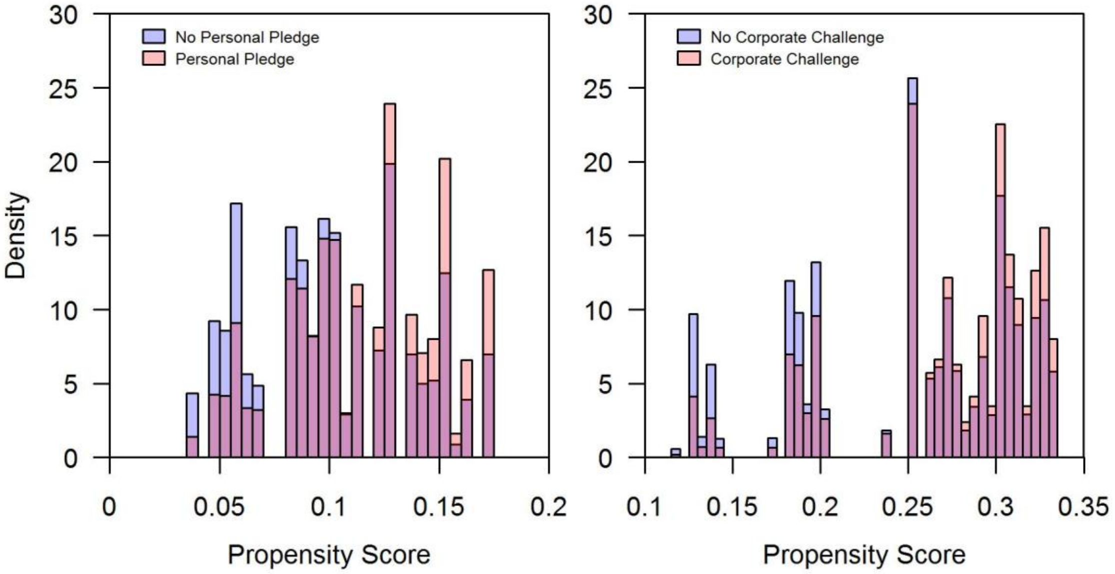

Web Figure 1. Propensity score distribution and overlap of participation in Personal Pledge and Corporate Challenge.

The lower bar indicates the overlap between the two groups. The upper bar indicates the excess density for the control group (blue) and intervention group (red).

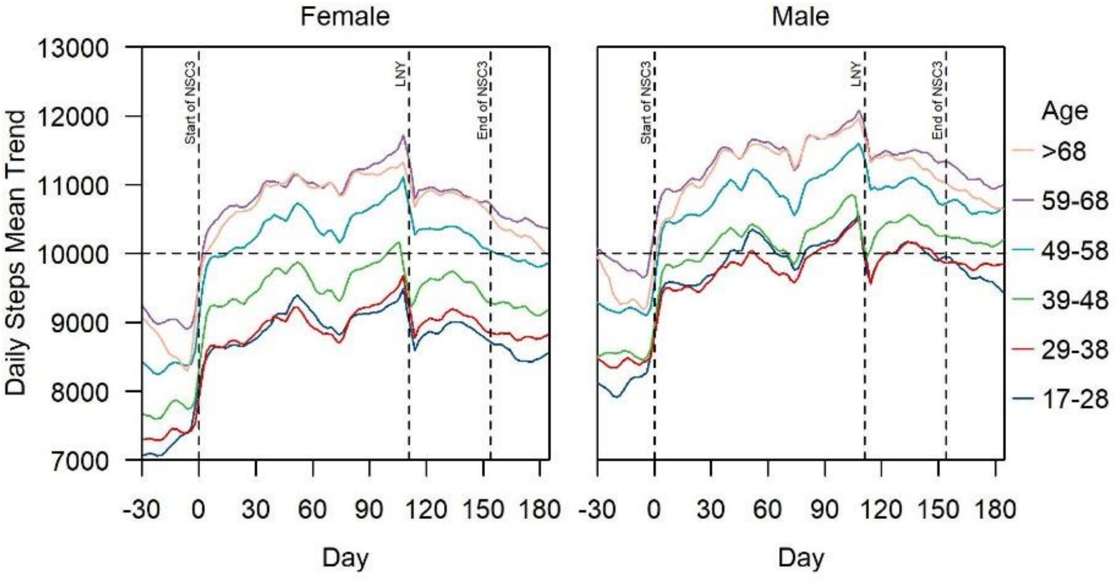

Web Figure 2. Daily Steps Mean trend of NSC3 sample from 28 September 2017 to 30 April 2018 (days -30 to 184), by gender and age groups.

The steps were decomposed into seasonal, trend and irregular components using locally estimated scatterplot smoothing (loess). (10) A seasonal frequency of 7 days were selected.

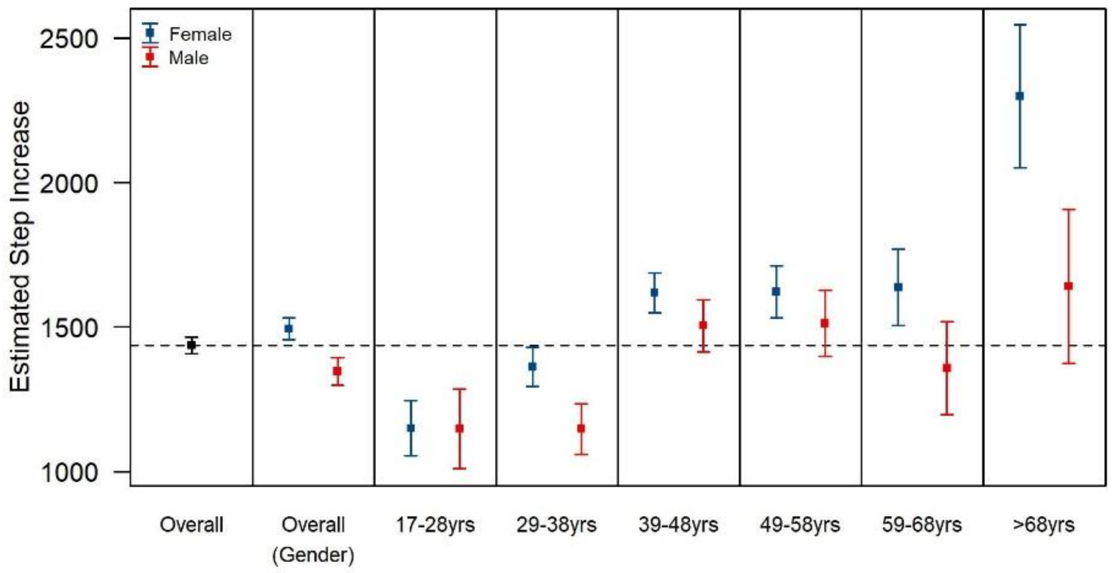

Web Figure 3. Estimated steps increase using regression discontinuity design of NSC3 from 28 September 2017 to 31 March 2018 (days -30 to 154), by gender and age groups.

The solid boxes in the middle of the error bar represent the mean; the "whiskers" represent the 95% confidence interval.

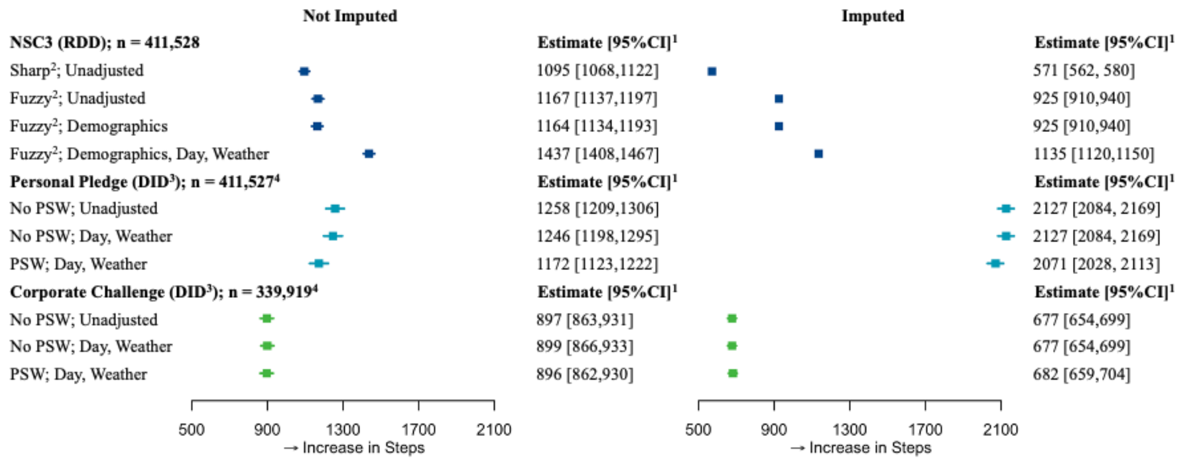

Web Figure 4. Sensitivity analysis 1: Estimated steps increase using regression discontinuity design of NSC3 daily step counts from 28 September 2017 to 31 March 2018 (days -30 to 154), difference-in-difference with fixed-effects regression of Personal Pledge daily step counts from 28 September 2017 to 31 March 2018 (days -30 to 154) and difference-in-difference with fixed-effects regression of Corporate Challenge daily step counts from 28 October 2017 to 30 April 2018 (days 0 to 184), adjusting for different covariates; with and without imputation.

RDD: Regression Discontinuity Design; DID: Difference-In-Difference; PSW: Propensity Score Weighted; Demographics: age group, sex, BMI group; Day: day of the week; Weather: maximum temperature, log (1 + rainfall).

1. 95% confidence intervals were constructed using standard errors clustered at the participant level.
2. Sharp RDD assumes that every participant signed up on the start date of NSC3, 28 October 2017 (Day 0). In contrast, fuzzy RDD relaxes this assumption by allowing participants to register at any time during the NSC3 intervention period.
3. Fixed-effects regression controls for all time-invariant variables, such as demographics.
4. One participant dropped as information if they took part in the Personal Pledge and Corporate Challenge was not available. An additional participant dropped for the Corporate Challenge as the registration date was after the start date.

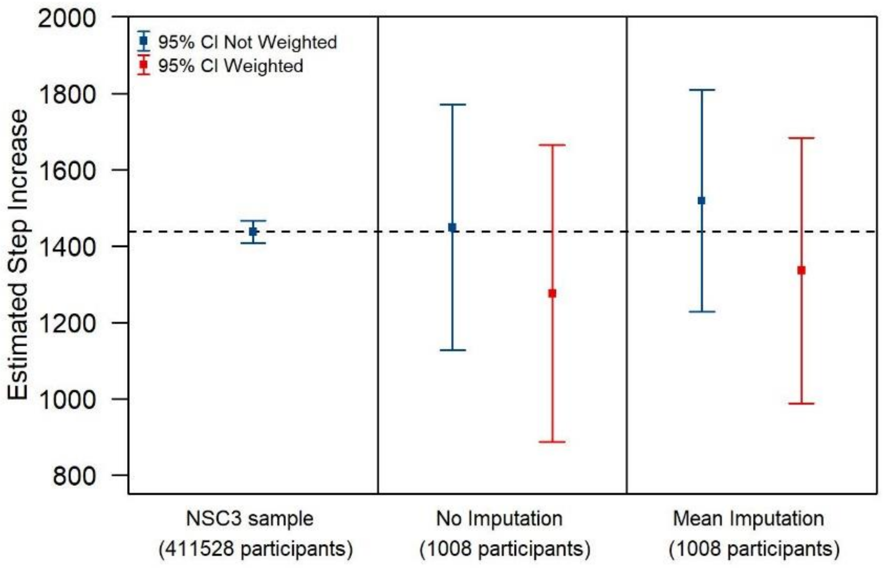

Web Figure 5. Sensitivity analysis 2: Estimated steps increase using regression discontinuity design of NSC3 from 28 September 2017 to 31 March 2018 (days -30 to 154) with and without mean imputation.

The solid boxes in the middle of the error bar represent the mean; the “whiskers” represent the 95% confidence interval. Twenty-one participants with at least three observations during the NSC3 pre-intervention period and at least 14 days of observations during the NSC3 intervention period were randomly selected from each subgroup to make up 1008 participants (21 x 2 (sex: male, female) x 4 (BMI: underweight, healthy, overweight, obese groups) x 6 (age groups: 17-28, 29-38, 39-48, 49-58, 59-68, >69 years). The red bars denote the estimates and confidence interval of the estimated step increase in steps by reweighing the observations based on their demographic proportions in the NSC3 sample.

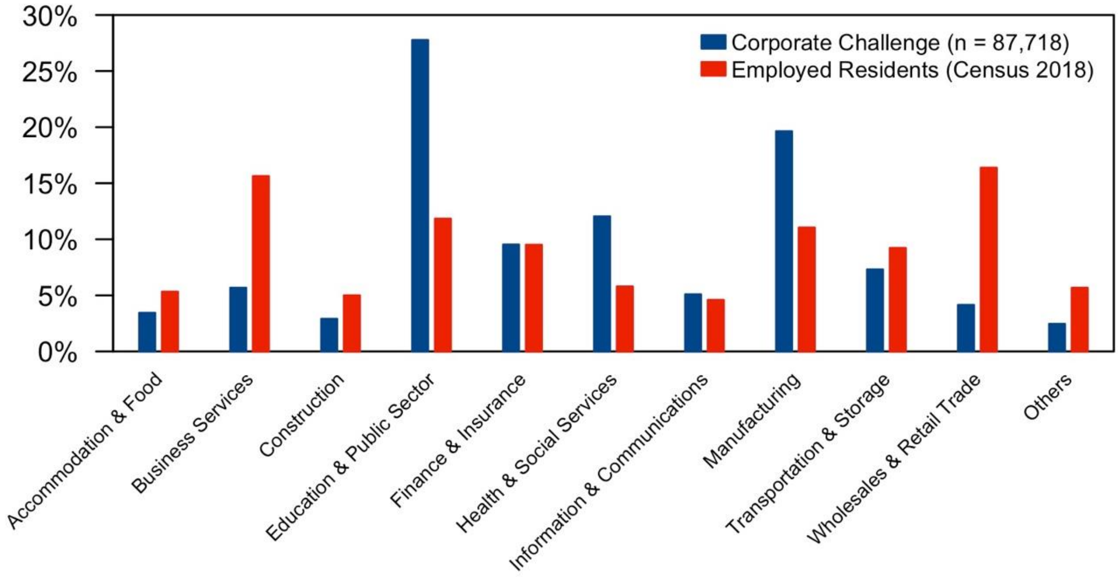

Web Figure 6. Comparison of industries between Corporate Challenge participants and employed Singapore residents aged between 25 to 64 years.

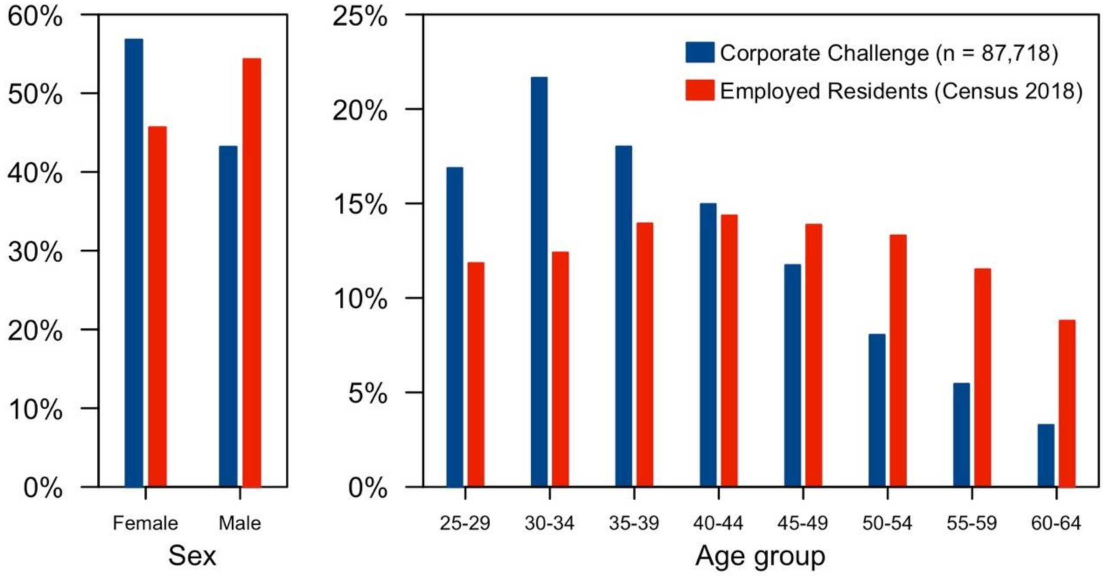

Web Figure 7. Comparison of sex and age group between Corporate Challenge participants and employed Singapore residents aged 25 to 64 years.

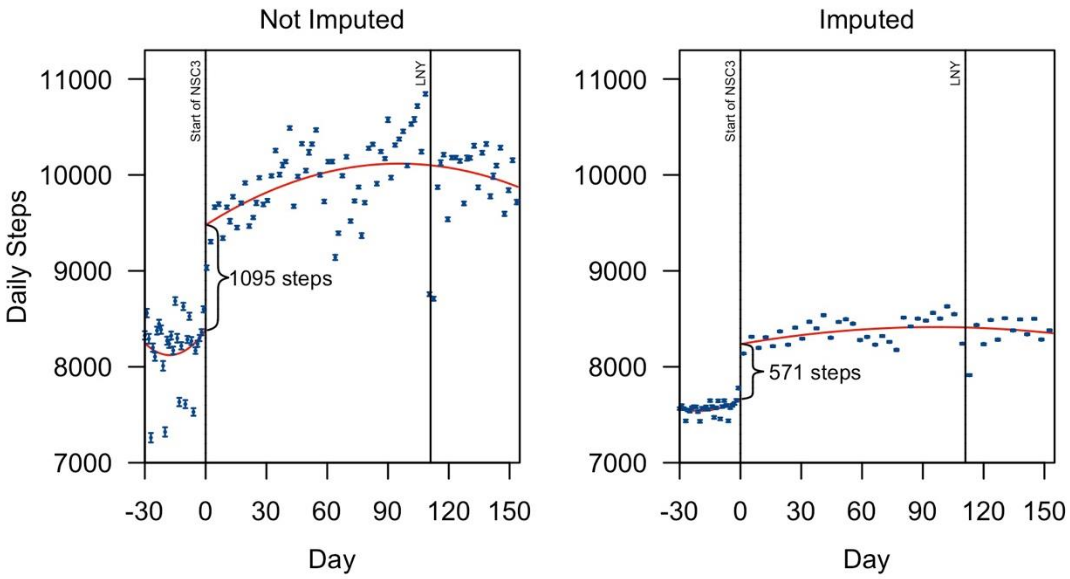

Web Figure 8. Sensitivity analysis 1: NSC3 regression discontinuity design plot of daily step counts with quadratic time trends from 28 September 2017 to 31 March 2018 (days -30 to 154), n = 411,528.

1. Observations are binned, and 95% non-parametric confidence intervals are computed (8). The blue boxes in the middle of the error bar represent the mean; the "whiskers" represent the 95% confidence interval. The red line is the prediction based on the mean from the sharp regression discontinuity design model.
2. NSC3: National Steps Challenge Season 3 (Started 28 Oct 2017, ended 31 Mar 2018 (days 0 to 154)); LNY: Lunar New Year (Started 16 Feb 2018, ended 17 Feb 2018 (days 111 to 112))

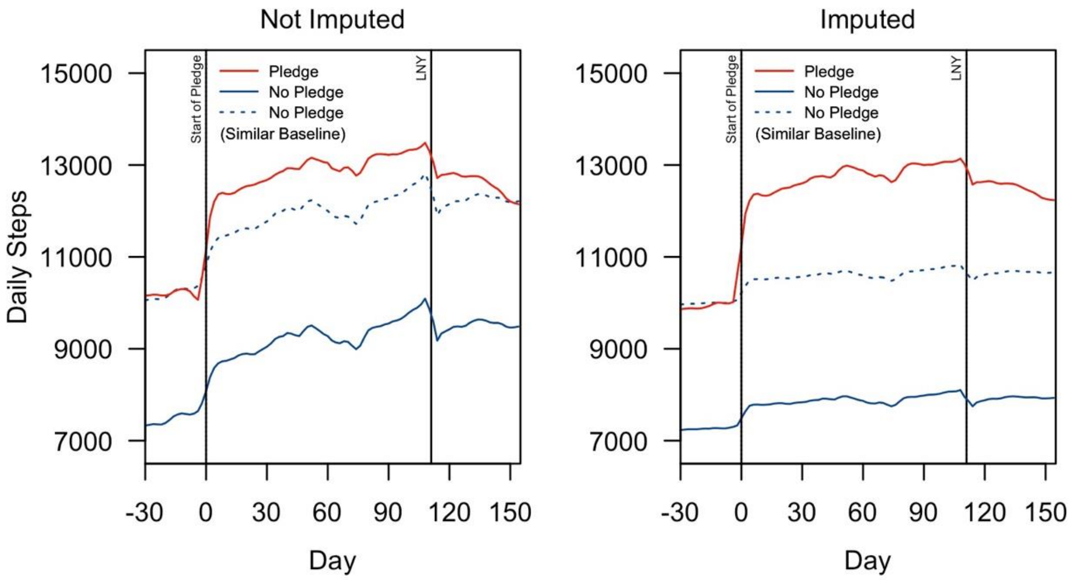

Web Figure 9. Sensitivity analysis 1: Daily Steps Mean trend of those in Personal Pledge and not in Personal Pledge from 28 September 2017 to 31 March 2018 (days -30 to 154), n = 411,527.

The solid blue line is the mean trend of the participants who did not participate in the Personal Pledge. The blue dotted line is the mean trend of the participants who did not participate in the Personal Pledge shifted downwards by the difference between the two means at the start of Personal Pledge. The increase in steps from Personal Pledge is the difference between the solid red line and the blue dotted line. LNY: Lunar New Year (Started 16 Feb 2018, ended 17 Feb 2018 (days 111 to 112)); Pledge: Personal Pledge (Started 28 Oct 2017, ended 31 Mar 2018 (days 0 to 154)).

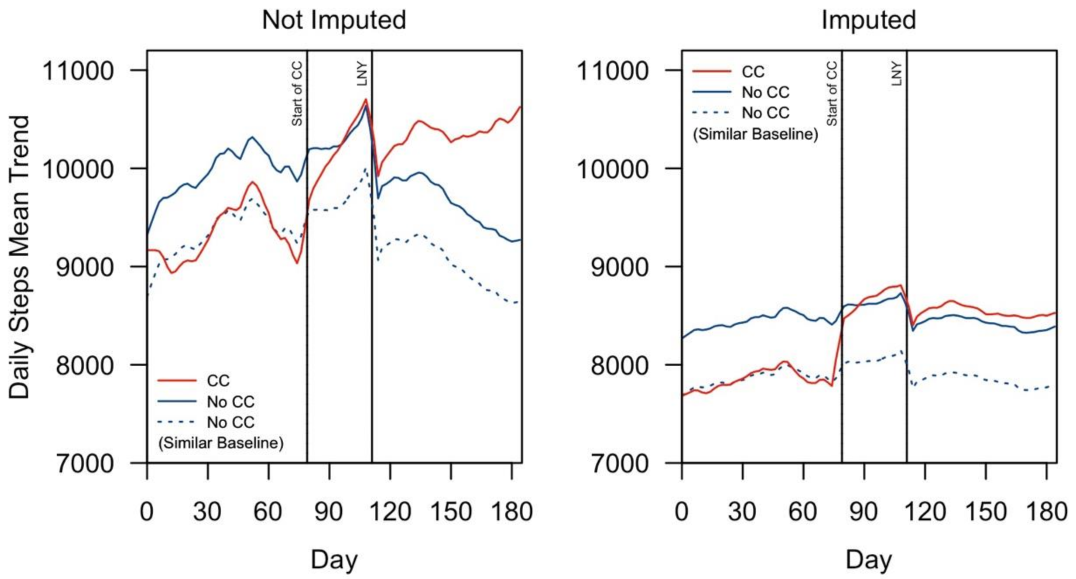

Web Figure 10. Sensitivity analysis 1: Daily Steps Mean trend of those in Corporate Challenge and not in Corporate Challenge from 28 October 2017 to 30 April 2018 (days 0 to 184), n = 339,919.

The solid blue line is the mean trend of the participants who did not participate in the Corporate Challenge. The blue dotted line is the mean trend of the participants who did not participate in the Corporate Challenge shifted downwards by the difference between the two means at the start of the Corporate Challenge. The increase in steps from Corporate Challenge is the difference between the solid red line and the blue dotted line.

LNY: Lunar New Year (Started 16 Feb 2018, ended 17 Feb 2018 (days 111 to 112)); CC: Corporate Challenge (Started 15 Jan 2018, ended 30 Apr 2018 (days 79 to 184)).

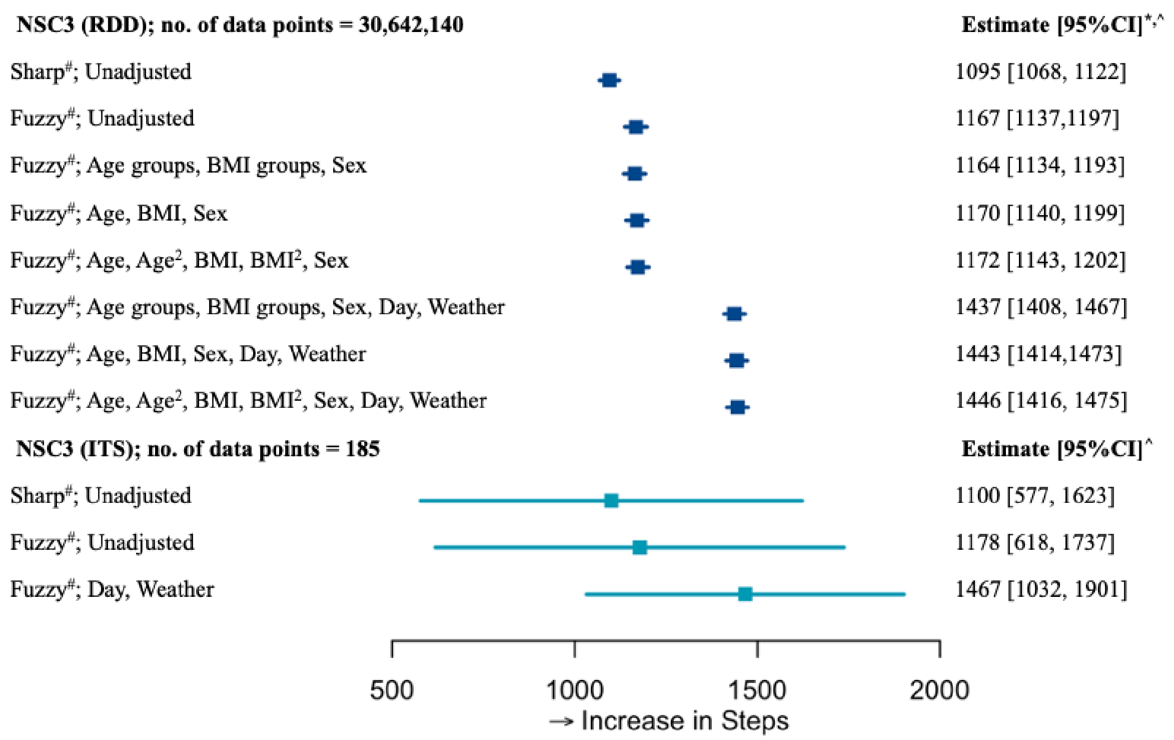

Web Figure 11. Comparison of estimated increase using regression discontinuity design using different covariates and interrupted time series of NSC3 step counts from 28 September 2017 to 31 March 2018 (days -30 to 154) (n = 411,528).

RDD: Regression Discontinuity Design; ITS: Interrupted time series; Day: day of the week; Weather: maximum temperature, log (1 + rainfall).

* 95% confidence intervals constructed using standard errors clustered at the participant level.

# Sharp RDD assumes that every participant signed up on the start date of NSC3, 28 October 2017 (Day 0). In contrast, fuzzy RDD relaxes this assumption by allowing participants to register at any time during the NSC3 intervention period.

^ The difference between ITS and RDD is that ITS uses the daily mean steps (we will only have 185 data points because our study period has 185 days, 1 for each day) while RDD uses the step counts from the participants (we will have 30,642,140 data points from 411,528 participants).

Fixed-effects regression controls for all time-invariant variables, such as demographics. Hence it does not matter if age and BMI are categorical or continuous.

References For Web Material

1. Health Promotion Board. *National Steps Challenge™ Season 3: Frequently Asked Questions.* Singapore: Health Promotion Board; 2017. (https://www.healthhub.sg/sites/assets/Assets/Programs/nsc-2017/National%20Steps%20Challenge%20Season%203_Main%20FAQs.pdf). (Accessed 21 June 2019).
2. Health Promotion Board. *Corporate Challenge Frequently Asked Questions (FAQs).* Singapore: Health Promotion Board; 2017. (https://www.healthhub.sg/sites/assets/Assets/Programs/nsc-2017/NSC%20FAQ%20for%20Corporate%20Challenge%202017%20%2824nov17%29.pdf). (Accessed 21 June 2019).
3. Blaga OM, Vasilescu L, Chereches RM. Use and effectiveness of behavioural economics in interventions for lifestyle risk factors of non-communicable diseases: a systematic review with policy implications. *Perspect Public Health* 2018;138(2):100-10.
4. Madley-Dowd P, Hughes R, Tilling K, et al. The proportion of missing data should not be used to guide decisions on multiple imputation. *Journal of Clinical Epidemiology* 2019;110:63-73.
5. Stan Development Team. RStan: the R interface to Stan. R package version 2.17.3. 2018.
6. Consultation WHOE. Appropriate body-mass index for Asian populations and its implications for policy and intervention strategies. *Lancet* 2004;363(9403):157-63.
7. Department of Statistics. *Labour*, *Employment*, *Wages and Productivity*. Singapore: Department of Statistics; 2022. (https://www.singstat.gov.sg/find-data/search-by-theme/economy/labour-employment-wages-and-productivity/latest-data). (Accessed 11 February 2022).
8. Calonico S, Cattaneo MD, Titiunik R. Robust nonparametric confidence intervals for regression-discontinuity designs. *Econometrica* 2014;82(6):2295-326.
9. Khandker SR, Koolwal GB, Samad HA. *Handbook on Impact Evaluation : Quantitative Methods and Practices*. 1st ed. Washington, DC: World Bank Publications; 2010.
10. Hyndman RJ, & Athanasopoulos, G. Forecasting: principles and practice. 1st ed. Melbourne, Australia: OTexts; 2018.
